# Fully bayesian longitudinal unsupervised learning for the assessment and visualization of AD heterogeneity and progression

**DOI:** 10.18632/aging.103623

**Published:** 2020-07-09

**Authors:** Konstantinos Poulakis, Daniel Ferreira, Joana B. Pereira, Örjan Smedby, Prashanthi Vemuri, Eric Westman

**Affiliations:** 1 Division of Clinical Geriatrics, Department of Neurobiology, Care Sciences and Society, Karolinska Institutet, Stockholm, Sweden; 2 Department of Biomedical Engineering and Health Systems (MTH), KTH Royal Institute of Technology, Stockholm, Sweden; 3 Department of Radiology, Mayo Clinic, Rochester, MN 55905, USA; 4 Department of Neuroimaging, Centre for Neuroimaging Sciences, Institute of Psychiatry, Psychology and Neuroscience, King’s College London, London, UK

**Keywords:** Alzheimer's disease, brain atrophy, neuroimaging, atrophy progression, longitudinal cluster analysis

## Abstract

Tau pathology and brain atrophy are the closest correlate of cognitive decline in Alzheimer’s disease (AD). Understanding heterogeneity and longitudinal progression of atrophy during the disease course will play a key role in understanding AD pathogenesis. We propose a framework for longitudinal clustering that simultaneously: 1) incorporates whole brain data, 2) leverages unequal visits per individual, 3) compares clusters with a control group, 4) allows for study confounding effects, 5) provides cluster visualization, 6) measures clustering uncertainty. We used amyloid-β positive AD and negative healthy subjects, three longitudinal structural magnetic resonance imaging scans (cortical thickness and subcortical volume) over two years. We found three distinct longitudinal AD brain atrophy patterns: one typical diffuse pattern (n=34, 47.2%), and two atypical patterns: minimal atrophy (n=23 31.9%) and hippocampal sparing (n=9, 12.5%). We also identified outliers (n=3, 4.2%) and observations with uncertain classification (n=3, 4.2%). The clusters differed not only in regional distributions of atrophy at baseline, but also longitudinal atrophy progression, age at AD onset, and cognitive decline. A framework for the longitudinal assessment of variability in cohorts with several neuroimaging measures was successfully developed. We believe this framework may aid in disentangling distinct subtypes of AD from disease staging.

## INTRODUCTION

Imaging biomarkers of brain morphology are increasingly used in research and clinical routine [1]. Dementia research has utilized such markers for the investigation of disease-related patterns from different cohorts [2, 3]. Structural neuroimaging markers are also used for selection of participants for clinical trials in Alzheimer’s disease (AD) [4]. The availability of longitudinal data provides us with the opportunity to assess changes over time. A new challenge for the imaging research community is the incorporation of longitudinal information in their study designs [5]. Some attempts to utilize these data to understand disease progression include the EuroPOND and TADPOLE projects (Links to Europond and TADPOLE https://tadpole.grand-challenge.org/ and http://europond.eu/software/). Other challenges include the assessment and fixation (ceteris paribus) of different study effects, the meaningful visualization of group differences and the simultaneous optimization of all these procedures for the sake of reproducibility in the presence of pragmatic sample sizes.

Unsupervised classification (clustering) is widely applied to neuroimaging data to unveil heterogeneous features within samples [3]. Several studies have investigated the heterogeneity in AD with the aim to define disease specific subtypes [6–13]. The clustering methods that are used today are mostly cross-sectional, in the sense that they only utilize baseline data. In the AD research field, many studies have focused on the unbiased identification of cortical and subcortical patterns of atrophy with structural MRI (sMRI). A recent study utilized longitudinal atrophy markers to find sets of brain regions with common progression patterns [14]. To date, no cluster-based study has included longitudinal data to identify groups of individuals with similar atrophy trajectories. Our current study intends to meet this necessity.

In cluster analysis, two approaches are widely used in the literature to account for or exclude the effect of confounders. The first approach is called the residual (de-trending) method [15, 16]. This approach applies a “correction” to the data with respect to a confounder that should not affect the results. The clustering algorithm is then applied to the de-trended data [10, 11, 17–19]. When using the de-trending approach, the statistical tests needed to be dramatically increased (one correction for each vertex/voxel/region of interest). Moreover, the cluster parameters are not optimized to the original data but given the artificial data (de-trended data). This can make the interpretation of results more difficult and introduce errors in reproducibility, since the results are based on a chain of procedures that are not connected in statistical terms. The second approach incorporates the effect that we want to account for in the analysis [9, 20]. This can be achieved with the addition of one fixed effect in the case of a statistical clustering model.

Another important feature of a neuroimaging clustering study is the comparison between the clusters obtained or the comparison between the clusters and a control group. This step is either incorporated in the clustering procedure, or it is performed as an independent post-clustering step. When this step is not included in the clustering procedure, we need to correct the resulting images for multiple statistical comparisons. This issue can be avoided in the case of simultaneous clustering and visualization.

Previous clustering studies grouped AD patients based on sMRI features from a single time-point [8, 10, 13, 17, 20–22]. Their conclusions were based on a single observation in time and the chance that those clusters reflect different stages of the disease and not specific patterns of atrophy (distinct AD subtypes) cannot be excluded. A longitudinal clustering design may help us disentangle disease stages from disease subtypes in a more reliable way. Finally, the longitudinal MRI data can be irregularly distributed between subjects. This needs to be accounted for in a model to obtain accurate estimates of atrophy progression.

In this study, we aimed to design and assess a framework for longitudinal clustering that incorporates: 1) simultaneous clustering of several longitudinal neuroimaging measures (multivariate data over time), 2) information for individuals with irregularly sampled observations, 3) comparison of the clusters with a control group, 4) the study and fixation (optional) of effects that should not drive the resulting clusters, 5) visualization of the resulting clusters for interpretation, 6) measures of uncertainty in the clustering. Our overall goal was to perform all the aforementioned methodological steps in one statistical model. The designed framework is applied to sMRI data of mainly Aβ positive AD patients and Aβ negative cognitively unimpaired (CU) subjects that were longitudinally followed up for 2 years (2-3 time points). To assess the results from the longitudinal clustering framework, we included all data with longitudinal information from our previous cross-sectional clustering study [13]. This allows us to compare the results from cross-sectional and longitudinal clustering in the same dataset. To be able to estimate cluster-specific atrophy trajectories is an important aspect that has been overlooked by cross-sectional AD subtypes studies [23]. This approach will provide relevant data to answer an unresolved question in the field, i.e., whether “AD subtypes” are truly distinct subtypes or just different groups of subjects at different stages of the disease.

## RESULTS

### Clustering evaluation

The reported results are based on 750 000 iterations with 500 iterations thinning where 250 000 iterations were the burn-in period, which therefore saved 1000 Markov Chain Monte Carlo (MCMC) samples. The distributions of the estimated parameters started converging after the burn-in samples and they remained stable thereafter for the remainder of the simulations. The deviance for the different models decreased with the increasing number of clusters (Supplementary Table 1). The different initializations produced various outputs from which the one with the packages’ default settings was the worst in terms of deviance. The model with initialization in the means of the clusters from our previous study [13] and the addition of uniform noise for eight clusters was optimal in terms of quality.

**Figure 1:**
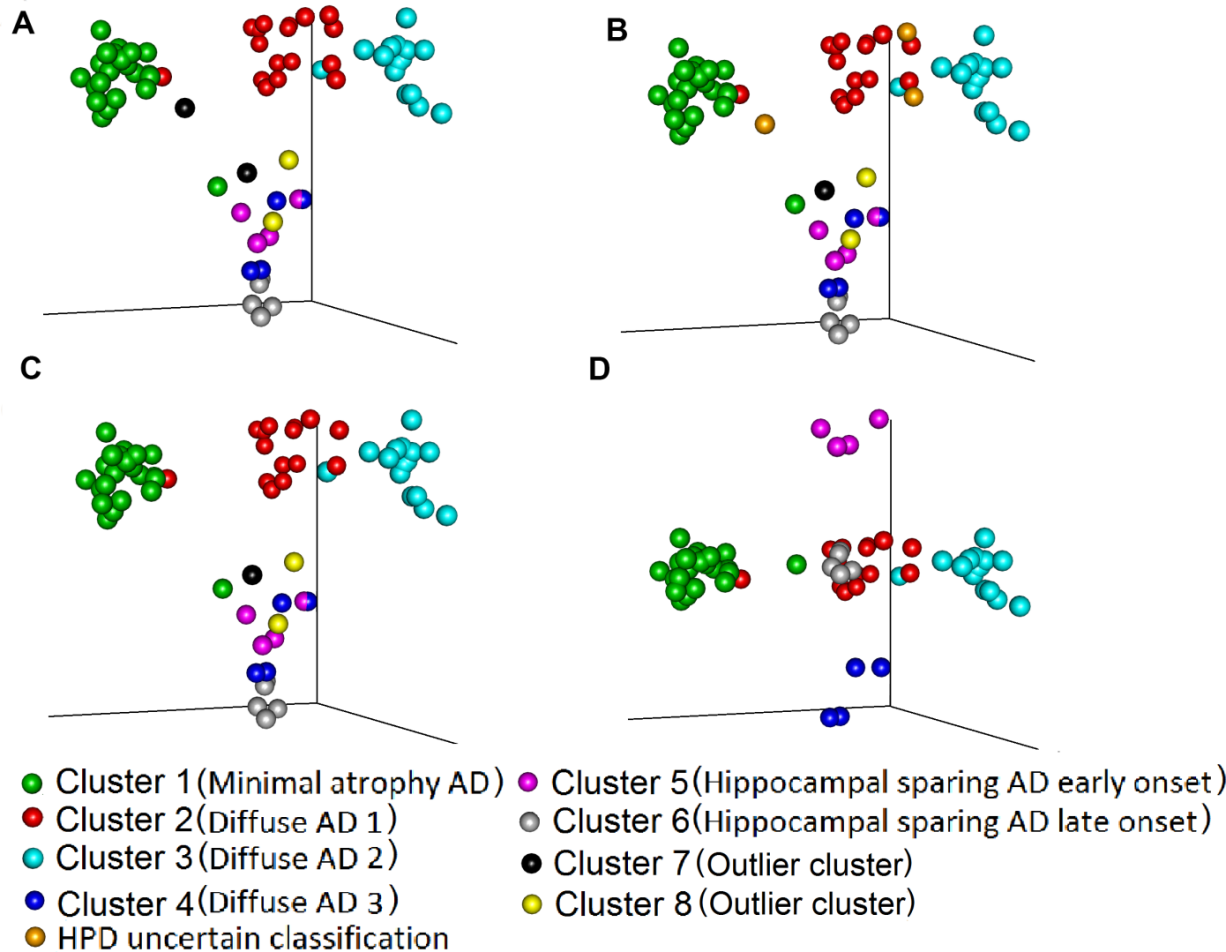
**Comparison of maximum probability and HPD interval classifications.** Three-dimensional representation of (Multidimensional scaled (MDS)) component-individual probabilities matrix (this matrix includes the probability of each subject being in any of the clusters). The scatter plots represent subjects and are coloured according to the clustering based on two approaches, maximum probability and highest posterior density intervals (HPD). (**A**) Subjects are coloured based on maximum probability classification (MDS components 1, 2 and 3). (**B**) Subjects are coloured based on HPD intervals classification. In comparison to **A**, in **B** we added the uncertain classification with orange colour (Two subject from cluster 2 and one subject from cluster 7 cannot be classified to any cluster with high certainty). (**C**) Colours are the same as in **B**, but we excluded from the plot the HPD uncertain classification subjects: orange and the outlier clusters 7: black and 8: yellow. (**D**) The subjects are coloured exactly as in **C** but the MDS components 1, 2 and 5 are plotted, to showcase the separation between cluster 4, 5 6. The names in parenthesis after the cluster numbers refer to Figure 2 and Table 2.

Figure 1 shows the multidimensional scaling coordinates of the component-subject probability matrix. Subjects are coloured dependent on the cluster to which they belong. Data from six subjects were excluded (Supplementary Figure 3 and Table 2): four subjects from the outlier clusters 7 and 8 based on the maximum probability rule (Figure 1A), and three subjects with uncertain classification with highest posterior density (HPD) intervals, analogous to confidence intervals in frequentist statistics (one subject from cluster 7 (already excluded) and 2 subjects from cluster 2) (Figure 1B). The remaining 66 subjects were used for further analysis. The separation between the six clusters in terms of probability for their subjects to belong to the same cluster is seen in Figure 1C, where clusters 1, 2 and 3 are clearly separated from each other. Visualization of the 1^st^, 2^nd^ and 5^th^ multidimensional scaled (MDS) components shows the separation between clusters 4, 5 and 6 (Figure 1D).

### Cluster characterization

**Figure 2:**
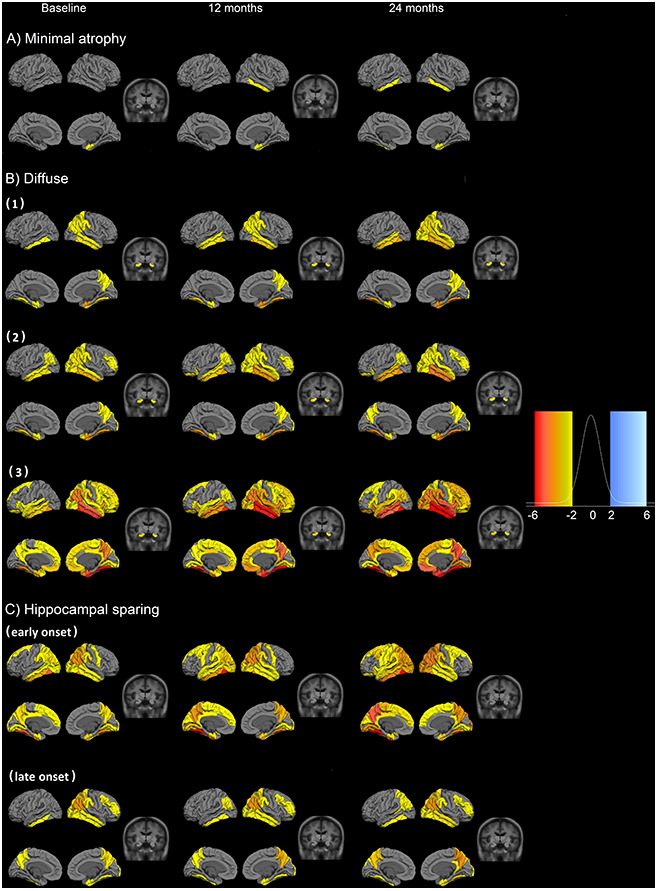
**Fitted values for cortical thickness and subcortical volumes for the different patterns of atrophy.** Atrophy fitted values of the six longitudinal atrophy patterns for the AD sample. Each row presents the median fitted values of the cortical and subcortical atrophy of the six components for three time points (baseline, 12 and 24 months from the first measurement). The data are presented as cognitively unimpaired group z-scores. (**A**) minimal atrophy pattern, (**B**) diffuse AD atrophy pattern, (**C**) hippocampal sparing AD atrophy pattern. Fixed effects: Intracranial volume = average Intracranial volume, Sex= female, Age = 75 years, Time from onset of dementia = 5 years, Education = 16 years, CSF Aβ1-42 = 100 pg/ml, CSF pTau 181P = 50 pg/ml. Data are presented as standard deviations below the estimated mean of the healthy cognitively unimpaired population.

Three main patterns of atrophy were found in the dataset: i) typical AD pattern (clusters diffuse 1, 2 and 3) (Figure 2B), ii) a minimal atrophy pattern (Figure 2A) and iii) a hippocampal sparing pattern (hippocampal sparing early and late onset) (Figure 2C).

The minimal atrophy cluster is characterized by initial atrophy in the entorhinal cortex and longitudinal thinning in adjacent inferior temporal gyrus (Figure 2A). The atrophy patterns in the three diffuse clusters (reported as typical AD) more closely follow the pattern of neurofibrillary tangles (NFT) spread as suggested by Braak and Braak [24]. However, differences between these three patterns do exist and may be attributed to age (even after correcting for this effect). The atrophy in the diffuse 3 cluster is more advanced (Figure 2B) and these subjects have lower cognitive performance (Supplementary Table 1). Two clusters are observed within the hippocampal sparing AD subtype. The degree of atrophy as well as the age at onset of dementia differentiate these two clusters (Figure 2C, Supplementary Table 1). The early onset hippocampal sparing cluster has a greater level of atrophy at baseline and accumulates atrophy faster over time, in contrast to the late onset hippocampal sparing cluster. In both hippocampal sparing clusters, the precuneus and the inferior parietal gyri are atrophied (Figure 2C). The 1^st^ and 3^rd^ quartile images show the dispersion around the mean cortical atrophy of each cluster (Supplementary Figure 1).

**Figure 3:**
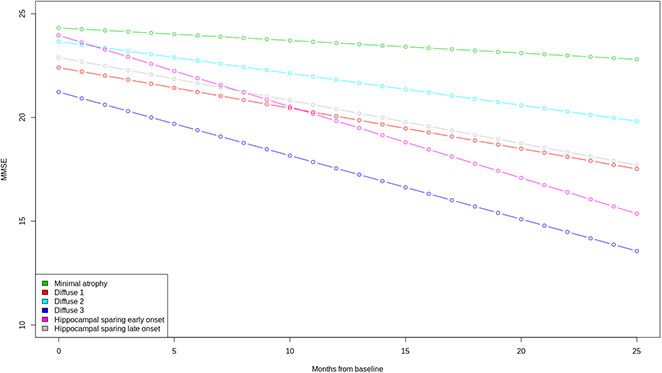
**Trajectories of MMSE total scores over time**. A mixed effect model estimated the MMSE total score differences between the six clusters at baseline and over time. Linear trend over time was assumed. Clear differences in the trajectories of MMSE were observed between the Minimal atrophy and Hippocampal sparing early onset/Diffuse 3 clusters. MMSE: Mini Mental State Examination.

The six clusters did not differ in terms of sex distribution, but they did differ in years of education (Supplementary Table 1). The lowest and highest median years of education are observed in the diffuse 2 cluster (12 years) and the hippocampal sparing early onset (18 years). The two clusters with hippocampal sparing patterns of atrophy differ in several aspects, such as the age of onset of dementia. The minimal atrophy cluster has the slowest decline over time in the clinical dementia rating scale (CDR), while the hippocampal sparing early onset cluster has the steepest decline (Supplementary Table 1). The hippocampal sparing early onset cluster also has a steep decline in constructional praxis, and the greatest deficits in ideational praxis at baseline. Although the minimal atrophy cluster has the best scores in all the Alzheimer's Disease Assessment Scale (ADAS) subscales at baseline, the hippocampal sparing late onset group has a better score in the word recognition task at baseline but declines very fast during the next two years. Total Mini mental state examination (MMSE) scores also differed between clusters (Figure 3). The minimal atrophy cluster had the highest baseline MMSE scores and maintained them during the available assessments. The diffuse 3 group had the lowest baseline MMSE scores and a steep decline. However, the steepest decline in MMSE was observed in the hippocampal sparing early onset cluster that started at levels comparable to the minimal atrophy cluster.

### Comparison to previous results

The subjects in the cross-sectional study [13] that were assigned to the diffuse 1 subtype are now distributed in more than one cluster with the highest prevalence in the diffuse 1 and 2 clusters (Table 1). Two subjects from the cross-sectional diffuse 2 cluster are now in the diffuse 3 and one in the outlier cluster 8. All the seven subjects from the cross-sectional hippocampal sparing subtype are still in the hippocampal sparing clusters. Three subjects assigned to the limbic predominant atrophy pattern in the cross-sectional study are now in the outlier cluster 7, diffuse 1 and the HPD uncertain group. The subjects in the minimal atrophy group are still mainly in minimal atrophy (17 subjects out of 20) while two subjects are assigned to the diffuse 1 cluster and one subject to the hippocampal sparing late onset cluster. Out of the four cerebrospinal fluid (CSF) Aβ1-42 negative AD subjects that are included in the current study, one subject is assigned to the longitudinal diffuse 2 cluster (was in the cross-sectional diffuse 1 cluster), one to the longitudinal outlier cluster 7 (was in the cross-sectional limbic predominant cluster) and two are assigned to minimal atrophy (both subjects were in the cross-sectional minimal atrophy cluster) (Table 2).

**Table 1:** Sample demographics.

	**AD patients**	**CU subjects**
N	72	31
Females N (%)	34 (47.2%)	15 (48.4%)
Age mean (sd)	76 (7.4)	74 (4.4)
Age at disease onset median(mad)	71 (8.9)	-
Years of education median(mad)	16 (3)	16 (3)
MMSE median(mad)	24 (1.5)	29 (0)
CDR global score median(mad)	0.72 (0.25)	0 (0)
ApoE e4 allele carrier N (%)	50 (69.4%)	3 (9.7%)
CSF Aβ_1-42_, median(mad)	137.38 (23.98)	234.11 (20.88)
CSF pTau _181_, median(mad)	37.5 (12.6)	18 (4.45)
ADAS word recall mean (sd)	6.17 (1.43)	2.81 (0.95)

Mad: maximum median distance, MMSE: mini mental state examination, ADAS: Alzheimer’s Disease Assessment Scale, CDR: Clinical Dementia Rating, CSF: cerebrospinal fluid. CSF values are in pg/ml. CU: cognitively unimpaired.

**Table 2:** Correspondence matrix.

	**Longitudinal clustering results**										
	**Names of clusters**	**Minimal Atrophy**	**Diffuse 1 (Typical AD)**	**Diffuse 2 (Typical AD)**	**Diffuse 3 (Typical AD)**	**Hippocampal sparing early onset**	**Hippocampal sparing late onset**	**Cluster 7**	**Cluster 8**	**HPD uncertain**	**Sum**
Cross-sectional clustering results	Diffuse 1 (Typical AD)	6	12	15	2	1	0	0	1	2	39
	Diffuse 2 (Typical AD)	0	0	0	2	0	0	0	1	0	3
	Hippocampal sparing	0	0	0	0	3	4	0	0	0	7
	Limbic predominant	0	1	0	0	0	0	1	0	1	3
	Minimal atrophy	17	2	0	0	0	1	0	0	0	20
	Sum	23	15	15	4	4	5	1	2	3	72

## DISCUSSION

The optimization of the longitudinal clustering model provided us with interesting findings that support its future use in imaging research for studying heterogeneity in healthy and pathological ageing. Clustering with several longitudinal measures that were irregularly sampled was successfully achieved. We incorporated information from a cognitively unimpaired sample to calculate age-corrected levels of atrophy, while avoiding the need to correct for multiple comparisons. Estimated subject-component probabilities made it possible to assess whether subjects are clustered with high certainty. All these features substantially help in the interpretation of the clusters. Moreover, the assessment of study effects within the model can assist in investigating which brain regions are statistically associated with them. The framework identified and characterized three distinct atrophy patterns with different trajectories over time and cognitive profiles.

The decision to start the algorithm optimization from the cross-sectional clustering results showed that when the algorithm is fed with initial information, the components are more meaningful, in the sense that almost all the components have some subjects in them. However, some of the cross-sectional clustering solutions were not optimal since they were not specifically adjusted to the dataset. We also checked that the variance of the posterior distribution of the fixed effects was considerably smaller than the large initial value to which that we set it [25].

This longitudinal clustering provides us with two additional types of information apart from the cluster assignment: 1) which subjects in a cohort are not well represented by one cluster (i.e. outliers), 2) which subjects are at risk of shifting from one cluster to another (i.e. HPD uncertain). When interpreting the data, we also considered two clusters as outliers. We decided that two subjects are too few to allow for an interpretation of the cluster characteristics and/or an extrapolation to the AD population. The estimated components should have a certain presence in the population in order to interpret them; otherwise the weakness of these clusters might introduce noise in the understanding of heterogeneity in the context of this application. Overall, the longitudinal clustering model combined with a priori chosen initial values for the cluster-specific parameters produced reasonable cluster estimates for meaningful interpretation of our longitudinal neuroimaging data.

The most typical AD like atrophy pattern is observed in the diffuse 1 cluster, that has all the demographic and cognitive characteristics of AD, such as the age of AD onset (>65 years of age), MMSE (18.5±7.1) and CDR global (1.3±0.8) [6, 7, 22, 26]. The diffuse 2 cluster is not substantially different in median fitted values from the diffuse 1 cluster. However, the higher age at onset (7 years older) and the percentage of females (53.5% in comparison to 46.7%) in the diffuse 2, together with the atrophy distribution dispersion in this cluster provided by the 1^st^ and 3^rd^ quartile atrophy maps (supplementary Figure 1), are somewhat reminiscent of the AD subtype known as limbic predominant AD [6, 7, 13, 17, 22, 27]. We speculate, given the longitudinal data and the previous cross-sectional study results [13], that the limbic predominant atrophy pattern is part of the AD disease staging rather than a distinct subtype. For some reason, this cluster has later onset, however patients seem to follow the Braak staging for neurofibrillary tangle (NFT) distribution and spread, hence they will likely develop typical AD at advanced stages. Regarding the diffuse 3 cluster, this is the most Correspondence between the assignments of subjects in the cross sectional clustering [13] and the current longitudinal study (clustering according to the highest posterior density intervals). The cross-sectional study clusters are in the rows and the longitudinal study clusters are in columns.

atrophied group of subjects in this dataset, its cognitive scores are very low and its frequency in the data is very small (4 subjects). Having already been reported in previous results of our group [13], we can now show that this group consists of subjects with already advanced atrophy at the time of the MRI. The model estimates a random intercept for each ROI at the time of the first MRI acquisition for each subject. Therefore, the subjects of the diffuse 3 cluster were separated from the other two diffuse atrophy clusters, since they had very low intercepts (great amount of atrophy) in the limbic areas and association cortex.

The minimal atrophy cluster, that includes subjects with high intercepts and small changes over time, is a cluster of considerable interest since the low amount of atrophy correlates well with the slow cognitive decline in this group. The frequency of minimal atrophy is higher than in previous studies [7, 13, 22], most probably due to the longitudinal design that allows subjects with slow cognitive decline to be followed up for a longer period. It has been proposed that tau-related pathophysiology and abnormal levels of Aβ alone without significant atrophy are enough to produce the dementia symptoms in the minimal atrophy subtype [22], perhaps through disruption of relevant brain networks in the absence of overt brain atrophy [28], in the context of lower cognitive reserve [28, 29].

The hippocampal sparing subtype with atrophy mainly in cortical areas has consistently been reported [6, 7, 13, 21, 22, 30]. Interestingly, our current study disentangled the observed hippocampal sparing pattern in two different clusters. A unique characteristic of the most atrophied hippocampal sparing group is the early onset as well as the high number of years of education, which is a proxy of cognitive reserve. This group seems to decline in cognition more rapidly than any other AD group, in agreement with the cognitive reserve hypothesis of faster disease progression in subjects with high reserve once a specific threshold has been reached [31]. In contrast, the less atrophied hippocampal sparing group has a late onset in the AD symptoms, which might be the reason for its less aggressive phenotype [32].

Regions that characterize the typical AD pattern of atrophy [6, 22] (inferior temporal and parahippocampal gyri, entorhinal cortex) are observed to be atrophied with high certainty as they are included both in 1^st^ and 3^rd^ quartile images for all three typical AD clusters (Supplementary Figure 1). In contrast, the precuneus and superior parietal gyri are included in both 1^st^ and 3^rd^ quartile images from the two hippocampal sparing clusters showing their importance in the identification of AD subtypes with regional atrophy markers. Finally, the minimal atrophy cluster has no significant regions of atrophy compared to the controls in the 3^rd^ quartile image which shows that even after considering the variability in the population atrophy patterns, the minimal atrophy cluster has its own profile separate from all the other subtypes.

There are also other aspects that differ between cross-sectional and longitudinal clustering. The statistical approach of the longitudinal clustering is based on distributional assumptions (each cluster has a multivariate normal distribution), while the cross-sectional clustering was distance-based. Therefore, the longitudinal model could accommodate fixed effects (variables that we want to account for), while the cross-sectional model could not (we de-trended these effects in advance). Another important methodological difference between the two approaches is the visualization of the clusters. The cross-sectional design included one more step after the clustering to compare AD groups with the sample of CU subjects in terms of regions of interests (p-value maps). Instead, the longitudinal model has an internal measure of similarity between AD groups and the CU sample, namely the fitted value maps where p-values are not calculated. We achieved a comparison between healthy ageing and AD clusters without overloading our dataset with statistical comparisons. More importantly, the level of difference in actual cortical thickness or volume between two clusters of subjects (fitted value) is easier to interpret biologically and clinically than the statistical differences between clusters of subjects (p-values).

Our study has some limitations. The sample size is limited for two main reasons. First, we wanted to use the results of our previous study as a ground truth for the clustering. Additionally, the exclusion criteria for CU subjects and AD patients were very strict, to ensure that the former group resembles a true sample of the healthy population over time, while the latter group had no missing information that can bias the interpretation of the results. This was intended to be a methodological study, although some biological interpretations have been made. Hence, for the methodological part we believe our current sample size is sufficient. Yet, it is our plan to replicate our current findings in a larger sample in the future to investigate the generalisability of the model in the AD population. In that study we will also estimate the normal ageing effect in atrophy using a longer follow-up than the one used here (24 months). Furthermore, the variable used as the time component in this study was the time from the first MRI acquisition, which helped the interpretation of the results in relation to the previous cross-sectional study, but it might limit the ability to assess whether a cluster of AD subjects reflects a distinct pattern of atrophy or a stage of the disease [22]. Our study has some strengths as well. We demonstrated that incorporating longitudinal information in the clustering of imaging data is possible. The analytical framework has successfully demonstrated its ability to identify outliers with dissimilar baseline and/or atrophy progression and set them in separate clusters. The method can also be applied it to different imaging modalities. The estimated model makes it possible to do two things that were not available before: 1) to estimate future levels of atrophy for any individual subject that belongs to the clusters (prognostic value) and 2) to estimate cluster assignment of new subjects that are not included in the model training (diagnostic value).

## CONCLUSIONS

In conclusion, a framework for longitudinal assessment of variability in cohorts with several neuroimaging measures was successfully tested and the results show that it can be used to understand complex processes in ageing and neurodegenerative disorders. To disentangle the complexity and heterogeneity, thus defining distinct subtypes of disease may lead to more personalized medicine in the future as well as to targeting the right populations for clinical trials.

## MATERIALS AND METHODS

### Participants

We used data from the Alzheimer’s disease neuroimaging initiative (ADNI), a project launched in 2004 in the US and Canada from Michael W. Weiner, MD. The initial goal of the ADNI-1 cohort was to gather neuroimaging data to better detect and track AD in its early stages. The inclusion criteria for AD patients were the following: 1) to fulfil the NINCDS/ADRDA probable AD criteria, 2) a CDR global score between 0.5 and 1, and 3) an MMSE total score between 20 and 26. The exclusion criteria for AD included: the use of psychotropic medication that could affect memory, history of significant head trauma, evidence of significant focal lesions at the screening MRI, and the existence of a significant neurological disease other than AD. For the CU subjects, inclusion criteria were an MMSE total score between 24 and 30 and a CDR global score equal to 0. Exclusion criteria for CU subjects comprised presence of depression, mild cognitive impairment (MCI) or dementia. For more information on the ADNI study, please see http://adni.loni.usc.edu/about/.

We included all subjects with longitudinal sMRI data and available CSF data (101 AD and 113 CU) from our previously published cross-sectional study [13]. In total, 75 subjects were excluded due to bad longitudinal image quality and processing results (see below). At baseline, 94% of the AD subjects were amyloid-beta (Aβ)1-42 positive, while only 31 CUs were included since we wanted them all to be negative for Aβ1-42 and phosphorylated tau (pTau). The cut-offs for Aβ1-42 and pTau used are discussed by Shaw et al. [33]. The CU sample was further limited by additional inclusion criteria: 1) remain as CU across all the available follow-ups and not only the follow-ups used in this study (0-36 months of continuous follow-up for the 31 CU subjects), 2) have longitudinal MRI for all the time points of the analysis.

Altogether, 104 individuals were included in the final analysis (Table 1), 72 AD patients (72 subjects had baseline and 12-month MRI scans, and 57 subjects had a 24-month MRI scan) and 31 CU (baseline, 12- and 24-month MRI scans).

### MRI acquisition and preprocessing

The MRI dataset consists of high-resolution sagittal 3D 1.5T T1-weighted Magnetization Prepared RApid Gradient Echo (MPRAGE) volumes (voxel size 1.1×1.1×1.2 mm3). Full brain and skull coverage were required and detailed quality control (QC) was applied to all the images [34].

Images underwent pre-processing with the longitudinal stream of FreeSurfer 6.0, where a subject-specific template is used [35]. For this study we utilized cortical thickness values for 34 cortical regions (Desikan-Killiany [36] atlas) and 7 subcortical volumes (hippocampus, amygdala, putamen, caudate, thalamus, accumbens, pallidum) from each hemisphere (Supplementary Table 2). The estimated total intracranial volume (eTIV) was also extracted for the statistical modelling of the volumetric data [37]. All data was processed through theHiveDB system [38]. The FreeSurfer output underwent visual QC and 29 AD and 48 CU subjects were excluded due to low output quality or since less than two continuous time points existed per subject after the QC.

### Statistical analysis

### Data standardization

The cortical thickness and subcortical volume ROI data of AD patients were standardized based on the sample of CU subjects, including mean centring and unit variance scaling. In this study, we adapted this longitudinal data procedure in order to account for the atrophy that is caused by the normal ageing process in the CU group over time. This ensures that the ageing time interval is accounted for. The z-values were calculated using the following formula $z_{j,t}^{i}=x_{j,t}^{i}-{\hat{\mu }}_{j,t}^{CU}\text{/}{\hat{\sigma }}_{j,t}^{CU},$ where *x* is the original measurement of subject i, in the time point *t* for the region *j*, while $\hat{\mu }$ and $\hat{\sigma }$ are the mean and standard deviation of the CU group at time *t* and region *j*. After this calculation, each value will resemble an atrophy level corrected for normal ageing levels and also normal decline over time, which was not previously done, and is crucial for biological and clinical interpretation of brain atrophy.

### Statistical longitudinal clustering

We used a generalized linear approach, which allows us to incorporate fixed and random effects that can serve in different ways in sMRI and other modalities. The algorithm clusters the random intercepts and slopes of each individual’s outcomes of interest (ROI measures in this study), with repeated measurements instead of repeated measurements data for each individual subject. A pair of subjects with similar estimated trajectories of atrophy (similar starting value/intercept and slope over time) are grouped together, while subjects with different trajectories are assigned to different groups. As previously discussed by Fraley et al. [39], if we know the number of clusters K, we can formulate the unobservable cluster allocation of subjects *i* as *P*(*U_i_* = *k*; *w*) = *w_k_*, k=1,..,K and i=1,..N. Here *w* is the vector of unknown cluster proportions that are positive and sum to 1. The meaning of *U_i_* is that *U_i_* = *k* when an observation ***Y**_i_* is produced by the model density *f_i_*_,*k*_(*y_i_*, *p_k_*, *p*), where *p_k_* are cluster specific parameters and *p* are population parameters. The marginal density of *Y_i_* is $\;f_{i}\left(y_{i};\theta \right)=\sum_{k=1}^{K}w_{k}f_{i,k}(y_{i},p_{k},p)$, where θ = {*w^T^*, *p_k_*, *p*} is the vector of unknown model parameters. Finally, clustering is based on the estimated values $\;{\hat{p}}_{i,k}\;$ that resemble the chances of an individual *i* belonging in cluster *k* [39].

The conditional mean response for each region j in a regression can be expressed as

$$E(Y_{i,j,l}\text{|}\beta_{j},b_{i,j})=\;x_{i,j,l}^{T}\;\beta_{j}+z_{i,j,l}^{T}b_{i,l},\;l=1,\ldots ,n_{i,j}$$ (1)

Here, *x_(i,j,l)_* are vectors of known covariates (fixed effects), *z_(i,j,l)_* is a vector that includes time values when the observations were made, *β_r_* is the vector of unknown fixed effect for region *j*,*b_j_* are i.i.d. random variables that express the *j_th_* response of subject i. A Gaussian distribution is used to model the ROI data (general linear model), but data of ordinal or nominal nature can be analyzed by changing the link function on the left part of the equation (1). For each individual i the conditional distribution of the joint random effects vector ***B**_i_*=(*B^T^_i,1_*,..,*B^T^_i,J_*) over *j* regions is multivariate normal,

$$B_{i}\text{|}\;U_{i}=k\;\sim N(\mu_{k},D_{k})$$ (2)

In (2), the *i_th_* subjects belong to the *k_th_* cluster (that is *U_i_=k*, for a *K* cluster solution), *μ_kis_* the unknown mean vector over *j* regions and *D_k_* is the cluster-specific positive definite covariance matrix. For each individual *i*, *Y_i,j,l_* (*j*=1,…,*J* and *l*=1,…,*n_i,j_*) are conditionally independent given the random effects ***B**_i_*. The random vectors *Y_1_*,…,*Y_N_*, as well as the random effect vectors ***B**_1_*,…,***B**_N_* that were mentioned above, are independent. In summary, the *μ_k_* and *D_k_*, comprise the cluster-specific parameters that we will estimate given the data to explore the various grey matter atrophy patterns. The dependence among the *Y_i,j_*s (different markers *j*) is included in the non-diagonal components of the matrix *D_k_*.

Accounting for external effects that might drive the resulting clusters within the model is convenient in this kind of analysis. Therefore, fixed effects *β_j_* in (1), common to all clusters (population level effects) are estimated for each of the external variables and brain region that we want to assess during the clustering analysis. Adding the regression dispersion parameters *ϕ_j_* to the *β_j_* vector, we summarise the model parameters common to all clusters. Those parameters are also assigned distributions. For more information specific to the distributions of those effects and their hyperparameters, please see the supplementary material (model specification).

This fixed effect approach allows for the fitting of the resulting cluster profiles (atrophy maps) for different combinations of fixed effects to investigate their regional contribution. Since the longitudinal data from almost all cohorts with MR acquisitions typically have different numbers of visits per subject (irregularly sampled), we chose a model that can utilize all available measurements of each individual subject to calculate regression intercepts and slopes (vectors *b_i,l_*). In the model, hierarchically centred generalized LMM are assumed with a non-zero unknown mean, *b_l_* for *l* = 1, … *L* regions (see formula 1). The model that combines all the aforementioned features, i.e., Multivariate Mixture of Generalized Mixed effect Models (MMGLMM) [25], is applied to longitudinal trajectories of atrophy (see packages “mixAK” and “coda”, R version 3.0.0 or higher). We chose a Bayesian approach for the clustering optimization because it includes prior distributions in the parameter estimates. This enables the algorithm to investigate the parameter space even in cases of small samples where likelihood information is limited.

The clustering algorithm estimates different outcomes. One outcome is the different cluster components. Each estimated multivariate Gaussian component resembles a pattern of atrophy that is observed in the dataset. Each individual subject is assigned a probability to belong to any of the components (soft clustering), rather than being assigned to a single component. The assignment of subjects into clusters is based on the maximum posterior probability rule (an individual is assigned to the component with the highest individual component probability). This is a much more realistic approach in comparison to hard clustering approaches used in most previous data-driven studies [7, 8, 10, 17, 30, 40], since the heterogeneity in AD is modelled here as a continuum and allows for mixed patterns instead of single patterns. Hence, the data-driven algorithm provides explicit information on whether a subject has a distinct atrophy pattern or a mixture of patterns through the estimation of subject component probabilities. The proposed framework clusters subjects of a cohort into groups (provides probability of subjects to belong in any of the clusters) and not patterns of atrophy into groups for a cohort (clusters of regions/vertices), as in the study by Marinescu et al. [14].

**Figure 4:**
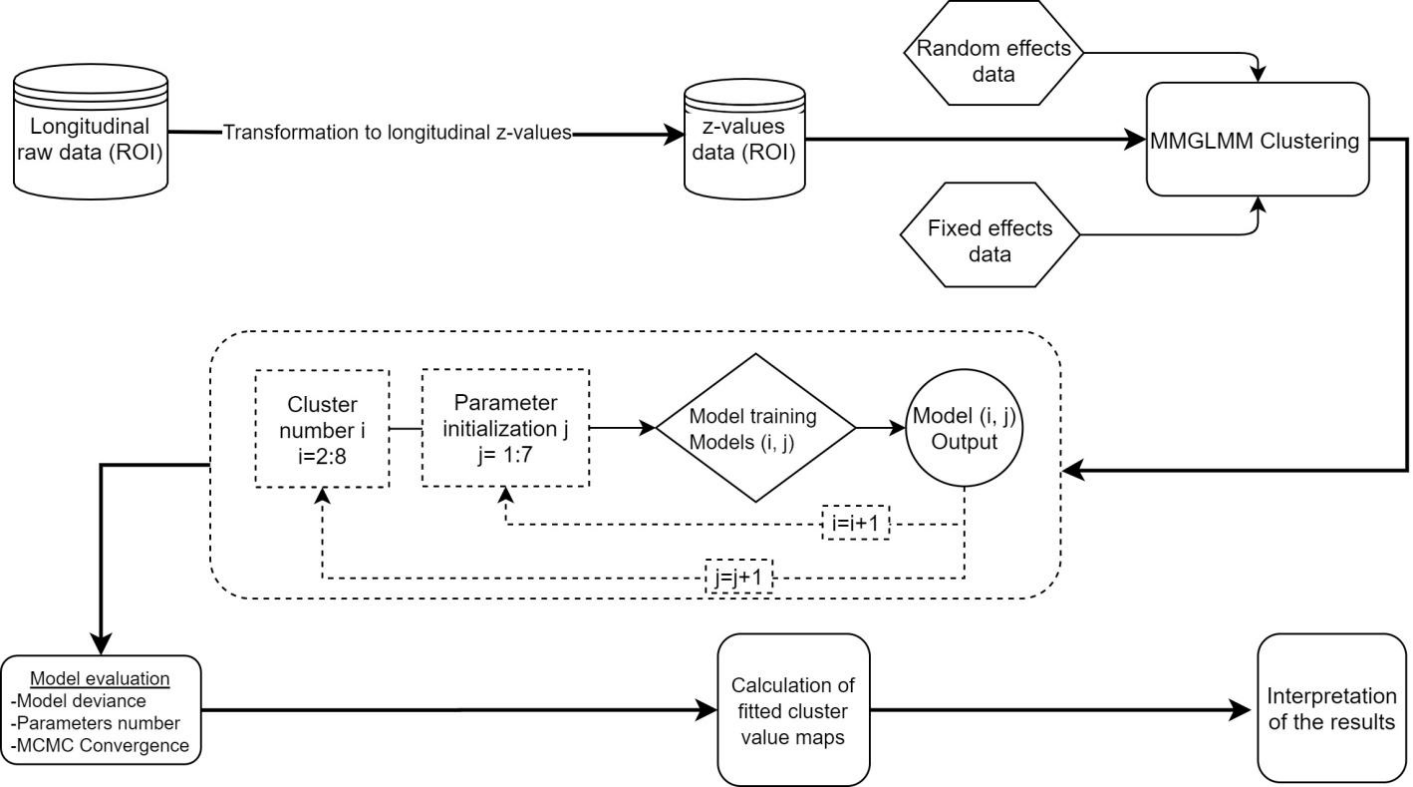
**Flowchart of the analysis.** The schematic representation of the analysis shows that all the steps after the data standardization are accomplished within the clustering and not in separate pipeline fashion like steps. ROI: region of interest, MMGLMM: Multivariate Mixture of Generalized Mixed effect Models.

A schematic representation of the proposed analytical framework is portrayed in Figure 4. The time from the first visit (baseline) was defined as a random effect for the sake of comparability with previous cross-sectional studies on AD subtypes [13, 17]. The intercept of the model will correspond to the atrophy levels on the first visit and the slope will show how these atrophy levels change over time. The fixed effects of the model are age, sex, education, years from the onset of dementia, total intracranial volume, and baseline CSF Aβ1-42 and pTau181 levels. The resulting clusters are visualized in terms of their fitted values on the median intercept (i.e. baseline), 12 months and 24 months after the baseline observation for a specific set of fixed effects. Only fitted values below two standard deviations of the CU mean are presented [41]. Measures of dispersion (1^st^ and 3^rd^ estimate distribution quartile) are also visualized in order to assess within-cluster variance. With those measures we can interpret how different the subjects within each cluster are. We also present the cortical maps of each individual and time point that was used in the analysis in the supplementary material to show how well the estimated components represent the individuals to which it is assigned.

The statistical model that we chose has all the features that were described above and its original specification can be found in [25]. The optimization was performed using the R language, version 3.4.1 [42]. The model is fully Bayesian and thus the output of the Markov chain Monte Carlo (MCMC) simulation is exploited to make inference on the population and cluster-specific parameters. To adequately explore the distributions of the estimated parameters and speed up convergence of the algorithm, we optimized the model from different initial values based on i) the packages’ default values [25], ii) previous study results [13] and iii) cross-sectional clustering on the baseline data including k-means clustering and hierarchical agglomerative clustering as well as the addition of uniform noise to increase randomness in the initialization [43, 44]. To identify the optimal solution for our dataset, we initially optimized models for 2-8 clusters for all the different initializations, summing to 49 MCMC chains. Then we assessed i) the model deviances (-2*logLikelihood) [25], ii) the quality of parameter convergence with respect to MCMC with high autocorrelation (visual inspection of the MCMC trace plots and auto-correlation values) [43] and iii) the quality of clustering with respect to observations with low classification certainty (See Supplementary Table 1). In our hybrid model evaluation approach, all three quality criteria were considered as important in the selection process (scaled to the same interval, 0-1) [45].

### Statistical comparisons

Annual changes in cognitive measures for the different clusters were estimated with linear regression. For the MMSE total score, trajectories of decline were also calculated for the clusters with a mixed effect linear regression. Fixed effects were used to assess the differences at baseline and in decline between the clusters and random effects accounted for the repeated measurements. Significance of fixed effects was not assessed due to sample size and the interpretation was based on the coefficient standard errors. The above-mentioned hierarchical model statistical analyses (comparison between clusters) were carried out using R 3.6.3 software.

## Supplementary Material

Supplementary Data

Supplementary Figures

Supplementary Table 1

Supplementary Table 2

Supplementary Tables 3 and 4
