# Supplementary material for: Fully bayesian longitudinal unsupervised learning for the assessment and visualization of AD heterogeneity and progression
Source: Aging (Albany NY). 2020 Jul 9;12(13):12622–47. doi: 10.18632/aging.103623 (PMC7377879; doi:10.18632/aging.103623)
Supplement: Supplementary Data [file aging-12-103623-s004..pdf]

SUPPLEMENTARY DATA

Model specification and optimization

Model specification

- Cluster-specific effect

The normally distributed random effects $\beta_{i,l}$ have parameters μ_k and D_k. The random parameter μ_k follows normal distribution with mean 0 and positive definite diagonal covariance matrix with 6 in the diagonal and 0 in the non-diagonal elements imposing independent apriori means. The inverted mixture covariance matrix D_k^{-1} follows independent Wishart distributions with 165 degrees of freedom and the diagonal covariance matrix with elements random parameters γ that follow gamma distribution with hyperparameters for shape = 0.2 and scale = 0.3. The a priori distribution for the proportions w_k is Dirichlet with parameters equal to 1.

- Population-specific effects

  - The population parameters β_j follow normal distribution with hyperparameter mean 0 and standard deviation 100. This is a relatively uninformative prior and we checked that the posterior standard deviations were much lower after being estimated with our data.

  - The regression dispersion parameters followed a gamma distribution (dispersion parameters often have either gamma or Wishart distribution since those distributions take only positive numbers, that is support over 0) with parameters for shape equal to 1 and the scale was a random hyperprior. The inverted hyperprior follows a gamma distribution too, with shape equal to 0.2 and scale selected by the routines of the package mixAK in R given our data.

Model optimization

Processing: The optimization process was longer and more intensive for larger numbers of clusters, since every additional component increased the number of new parameters to be estimated. The cluster-specific parameters (random effects) such as the mean, covariance matrix and proportion of cluster parameters were the most demanding parameters to optimize, especially in the case of 7 and 8 cluster solutions. The visual inspection of the MCMC trace plots for these parameters showed large steps at the first thousand iterations (burn in period and some iterations later) and then a stable distribution (good chain mixing) is produced. Initially, the packages' default values for the parameters were used to see the extent of adaptation of the model to the data without any help of locally optimal solutions. The results showed that the model tends to produce 1-2 components that represent the actual dataset, while the rest of the components have non-sensible values. Moreover, the subjects were classified with high certainty in these 1-2 realistic components. This is advantageous because it means that the probabilistic clustering correctly identifies the components that represent the data in the best way. However, the rest of the components remained empty, which is a sign that the algorithm estimates components with zero presence in the dataset if it is not given some hints on where the data actually lie in the parameter space. The model with default initial values was not considered adequate to describe the dataset since too many parameters had no meaning in our application.

- Outlier clusters: The first outlier cluster includes one subject who is characterised by little bilateral temporal atrophy as well as subtle right hippocampal atrophy at 12 months, that cannot be captured at the 24-months observation. This may be a matter of longitudinal data preprocessing deviance in the volume estimation. The second outlier cluster has two subjects with typical AD cortical atrophy. However, one of them has no subcortical atrophy and the other has subtle left hippocampal atrophy that cannot be captured at the 12-moths assessment, together with large bilateral caudate volumes (in all timepoints) in comparison to the CU sample.

- Composite quality measure: The idea behind calculating a composite measure of model quality was inspired by the fact that all chains converged perfectly for none of the models. However, some autocorrelation was allowed to exist, which often happens in applications of Bayesian statistics[1]. We accepted a certain extent of autocorrelation within chains but did not accept any solution with high values[2]. The number of chains that had some autocorrelation among the random effects of the selected model was only 6% of the overall

[1]Gelman A, Carlin JB, Stern HS, Dunson DB, Vehtari A, Rubin DB. Bayesian data analysis. Chapman and Hall/CRC; 2013. Chapter 11.
[2]Dobson AJ, Barnett AG. An Introduction to Generalized Linear Models. CRC Press; 2018. Chapter 13.

parameters, which is a reasonable amount (considering that the chains are generally mixing sufficiently well). Criteria such as Akaike's information criterion and Bayesian information criterion provide information about deviance, parameter number and sample size, but disregard uncertainty in the model parameters[3]. In our approach, we used information about uncertainty and quality of Bayesian optimisation together with the deviance, to exploit the quality of the deeper features of our model structure.

- No initial random slope information: The lack of initial values for the slopes of each cluster (we set the initial slopes to zero due to lack of longitudinal cluster information) might be the reason behind the superiority of a solution with the introduction of uniform noise. In this way, we let the algorithm search for an optimal solution that may not fit (in the parametric space) exactly to the previous study's solution but in a parametric region close to it. Thus, we give more flexibility to the optimizer of the model to end up in the same values (as the cross-sectional study), only if these are the optimal ones. In this way, w avoid stumbling on a local optimum.

[3]Bishop CM. Pattern Recognition and Machine Learning (Information Science and Statistics) [Internet]. 2007. Page 33.
