# Supplementary material for: Fully bayesian longitudinal unsupervised learning for the assessment and visualization of AD heterogeneity and progression
Source: Aging (Albany NY). 2020 Jul 9;12(13):12622–47. doi: 10.18632/aging.103623 (PMC7377879; doi:10.18632/aging.103623)
Supplement: Supplementary Figures [file aging-12-103623-s005..docx]

SUPPLEMENTARY FIGURES


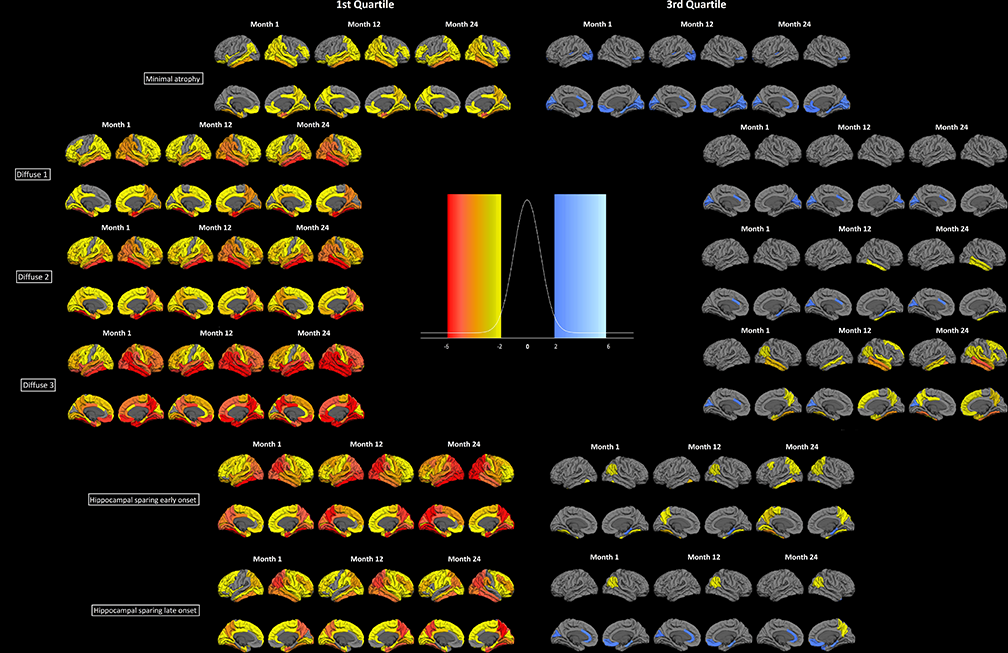


Supplementary Figure 1. 1^st^ and 3^rd^ quartile fitted value cortical thickness images. Fitted values of the 6 longitudinal atrophy clusters for the AD sample. Each row presents the quartile 1 and quartile 3 fitted value images of the cortical and subcortical atrophy of the 6 clusters for three time point (1, 12 and 24 months from the first measurement). The data are presented as controls z-scores. Fixed effects: Intracranial volume = average Intracranial volume, Sex= female, Age = 75 years, AD duration = 5 years, Education = 16 years, CSF Abeta 42 = 100 pg/ml, CSF Ptau181P = 50 pg/ml. These images help to understand and characterize each cluster


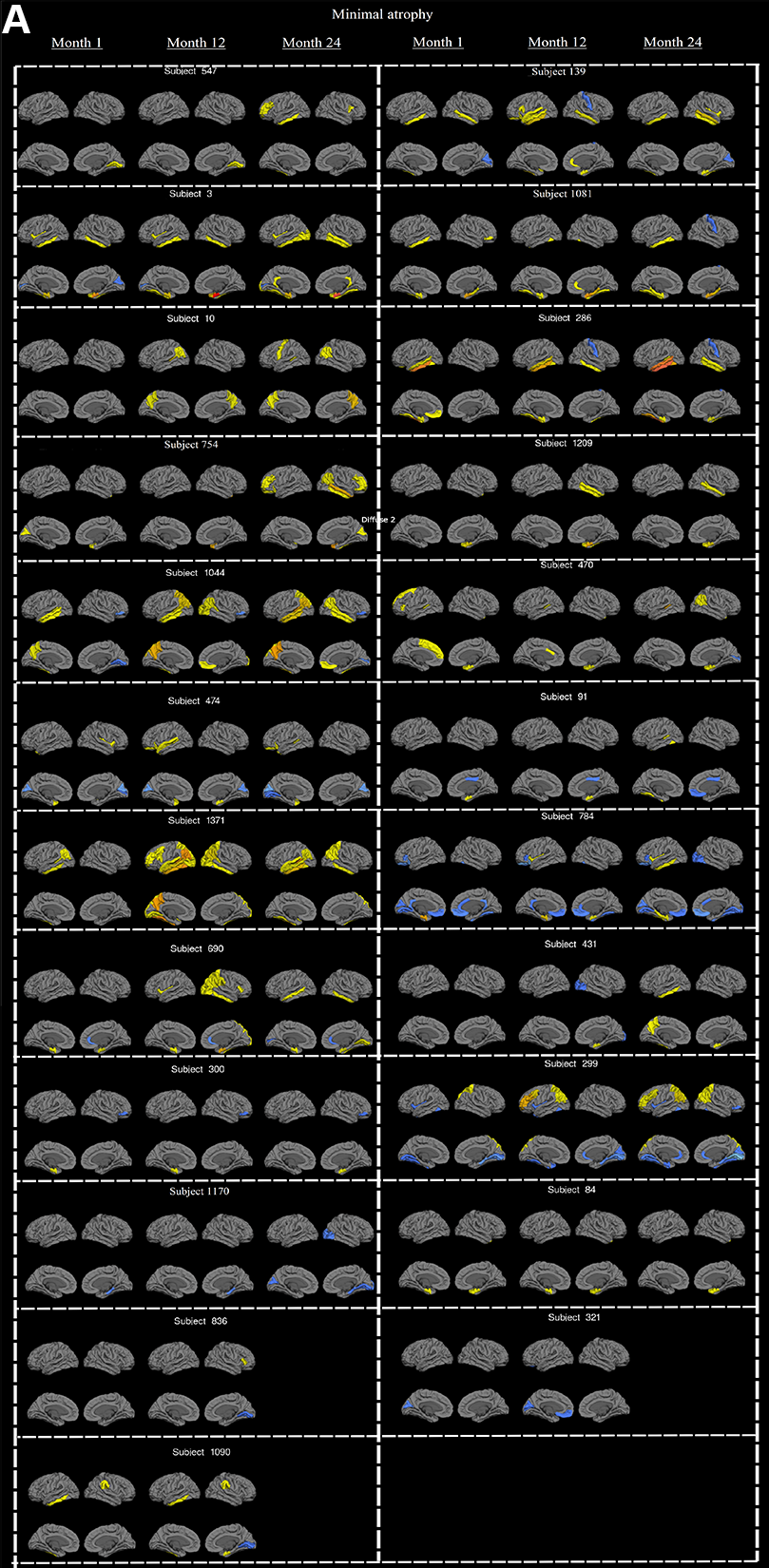


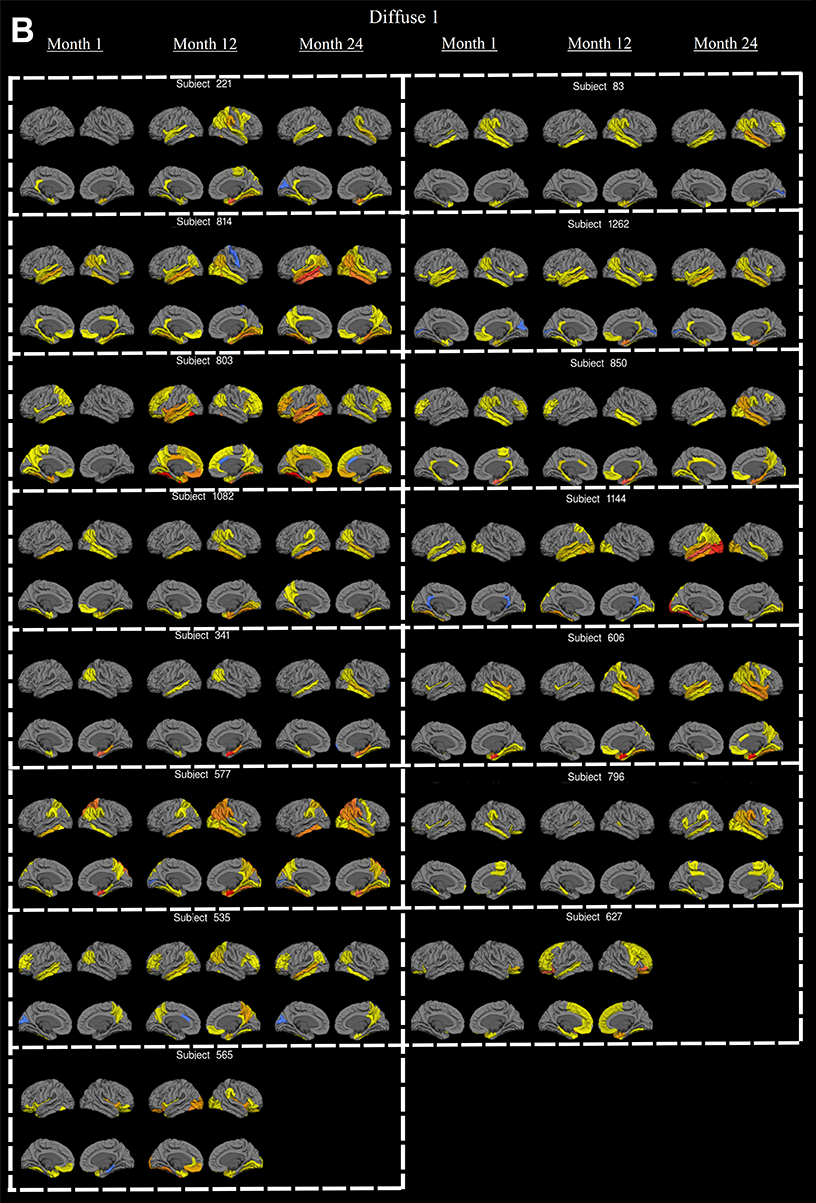


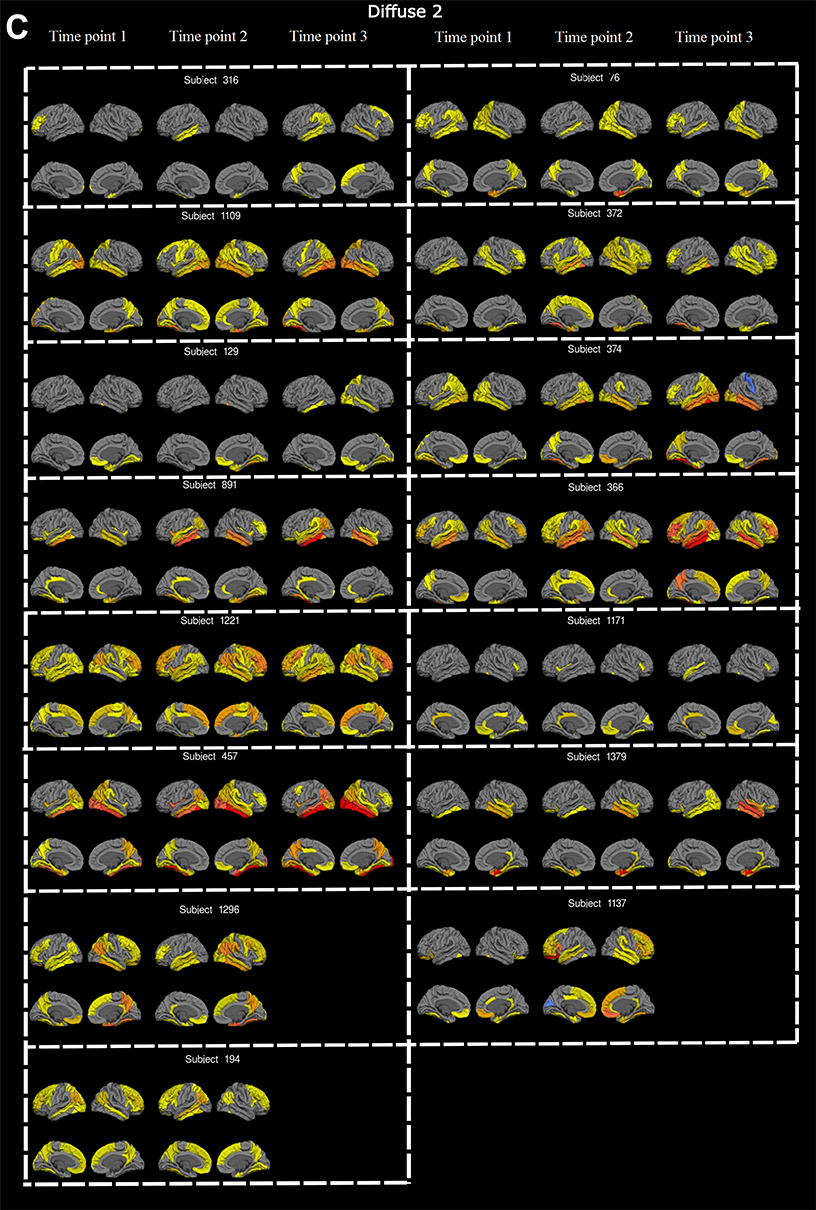


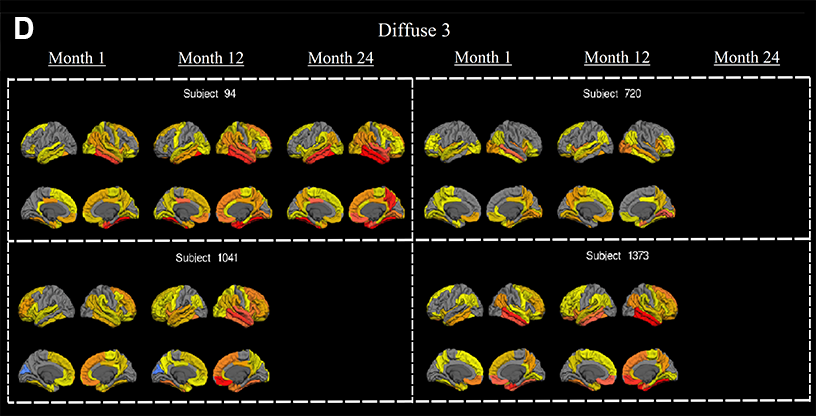


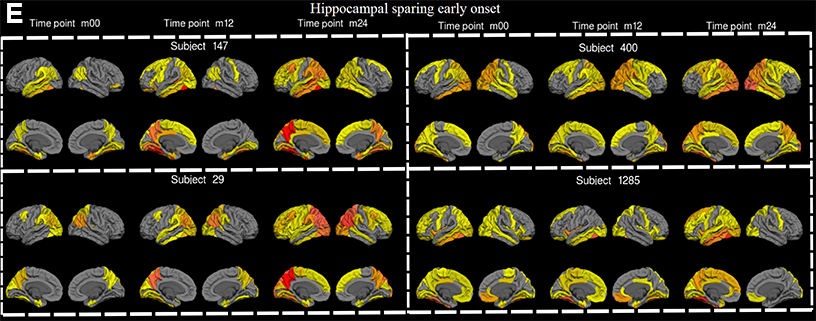


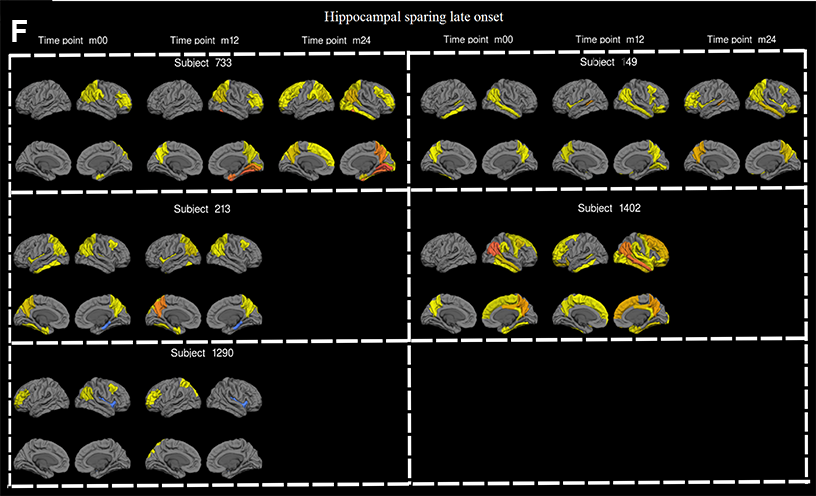


Supplementary Figure 2. Individual cortical atrophy pattern for each cluster’s input data. Minimal atrophy. This is a visualization to increase the transparency of the clustering procedure. Each cell (top row: subject ID) comprises the cortical thickness of one subject for their different time points (second row, Time point m00: baseline observation, Time point m12: 12 months follow up, Time point 24: 24 months follow up). The subject atrophy maps are presented in terms of z-values after a linear correction for the same effect variables as the clustering algorithm fixed effects. The legend of colours is the same as in the Supplementary Figure 1. Only thickness that exceeds 2 standard deviations from the control group regional distributions are presented. (A) Minimal atrophy (B) Diffuse 1, (C) Diffuse 2, (D) Diffuse 3,
(E) Hippocampal sparing early onset, (F) Hippocampal sparing late onset.


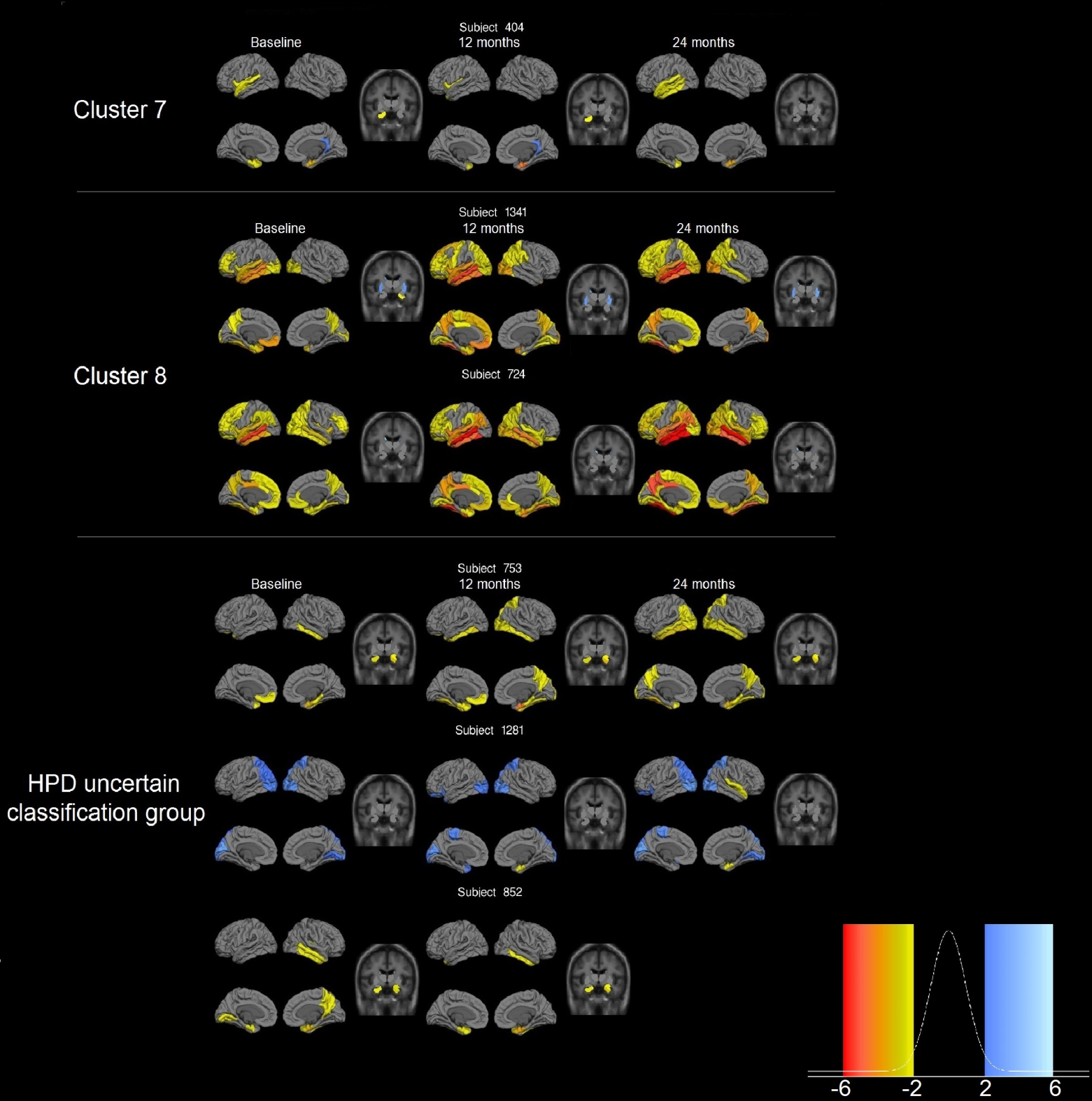


Supplementary Figure 3. Demographics of outlier clusters and HPD uncertain group. The individual subject images have the same colour legend as in Supplementary Figure 1 and therefore are presented in terms of standard deviations above or below the average of the control group. The outlier cluster 7 consists of one subject, the cluster 8 of 2 subjects and the HPD uncertain classification group of 3 subjects. The cluster 1 and cluster 7 are outlier clusters, while the HPD group subjects are subjects that could not be classified with high certainty in any of the 6 main clusters of the analysis result.
