# Supplementary material for: Fully bayesian longitudinal unsupervised learning for the assessment and visualization of AD heterogeneity and progression
Source: Aging (Albany NY). 2020 Jul 9;12(13):12622–47. doi: 10.18632/aging.103623 (PMC7377879; doi:10.18632/aging.103623)
Supplement: Supplementary Table 1 [file aging-12-103623-s001..docx]

Supplementary Table 1. Demographic and clinical characteristics of the clusters.

|  |  | Minimal atrophy | | Diffuse 1 (Typical AD) | | Diffuse 2 (Typical AD) | | Diffuse 3 (Typical AD) | | Hippocampal sparing early onset | | | Hippocampal sparing late onset | |
| --- | --- | --- | --- | --- | --- | --- | --- | --- | --- | --- | --- | --- | --- | --- |
|  | | m0 | m24 | m0 | m24 | m0 | m24 | m0 | m12 | m0 | m24 | | m0 | m24 |
| Demographics | |  | | | | | | | | | | | | |
| N (%) | | 23 (35%) | 20 |  | 13 | 15 (23%) | 12 | 4 (6%) | 4 (6%) | 4 (6%) | | 4 (6%) | 5 (7%) | 2 |
| Females N(%) | | 9 (39.1%) | 9 (45%) | 7 (46.7%) | 6 (46.2%) | 8 (53.3%) | 6 (50%) | 2 (50%) | 2 (50%) | 2 (50%) | | 2 (50%) | 2 (40%) | 1 (50%) |
| Age | | 76 (10.4) | 78 (12.6) | 74 (5.9) | 76 (5.9) | 79 (3) | 81 (3.7) | 73.5 (3.7) | 73.5 (3.7) | 66.5 (6.7) | | 66.5 (6.7) | 70 (14.8) | 74.5 (23) |
| Age disease onset | | 70 (10.4) | 71.5 (11.9) | 68 (7.4) | 72 (7.4) | 75 (4.4) | 73.5 (5.2) | 68.5 (5.2) | 68.5 (5.2) | 59.5 (3.7) | | 59.5 (3.7) | 67 (11.9) | 70 (23.7) |
| Years of education | | 16 (4.4) | 15 (3.7) | 14 (3) | 14 (3) | 12 (5.9) | 13.5 (3.7) | 16 (3) | 16 (3) | 18 (0) | | 18 (0) | 16 (3) | 15 (1.5) |
| Apoe e4 allele carrier N(%) | | 18 (78.3%) | 15 (75%) | 12 (80%) | 10 (76.9%) | 9 (60%) | 8 (66.7%) | 3 (75%) | 3 (75%) | 2 (50%) | | 2 (50%) | 2 (40%) | 1 (50%) |
| CSF biomarkers | |  | | | | | | | | | | | | |
| Aβ1-42 | | 129.81 (17.57) | 129.38 (16.54) | 136.83 (41.35) | 140.09 (27.1) | 140.37 (25.43) | 153.81 (11.4) | 153.81 (11.4) | 153.81 (11.4) | 128.8 (17.09) | | 128.8 (17.09) | 143.32 (5.69) | 145.71 (9.24) |
| pTau 181P | | 38 (13.34) | 37.5 (14.83) | 44 (20.76) | 44 (20.76) | 35 (5.93) | 35.5 (5.93) | 35.5 (5.93) | 35.5 (5.93) | 36.5 (14.08) | | 36.5 (14.08) | 50 (35.58) | 36 (20.76) |
| Cognitive measures | |  | | | | | | | | | | | | |
|  | | Median (mad) | Annual change (se) | Median (mad) | Annual change (se) | Median (mad) | Annual change (se) | Median (mad) | Annual change (se) | Median (mad) | Annual change (se) | | Median (mad) | Annual change (se) |
| MMSE | | 25 (1.5) | -0.8(0) | 22 (1.5) | -2.2(0.1) | 24 (1.5) | -1.9(0) | 21.5 (0.7) | -3.3(0.1) | 25.5 (0.7) | -4.1(0.1) | | 23 (1.5) | -2.6(0.1) |
| CDR global | | 0.65 (0.24) | 0.15(0) | 0.77 (0.26) | 0.27(0.01) | 0.7 (0.25) | 0.23(0.01) | 0.88 (0.25) | 0.25(0.02) | 0.75 (0.29) | 0.37(0.01) | | 0.6 (0.22) | 0.22(0.01) |
| ADAS 11 | |  | | | | | | | | | | | | |
| ADAS Q1 Word recall | | 5.3 (1) | 0.5(0) | 7 (1.5) | 0.2(0) | 6.3 (1) | 0.4(0) | 8.3 (1) | 0.7(0) | 6.7 (2.7) | 0.7(0.1) | | 6.3 (1) | 0.5(0) |
| ADAS Q2 Commands | | 0 (0) | -0.1(0) | 0 (0) | 0.2(0) | 0 (0) | 0.1(0) | 1 (0) | 0.3(0) | 0 (0) | 0.6(0) | | 0 (0) | 0.2(0) |
| ADAS Q3 Constructional praxis | | 1 (0) | 0.1(0) | 1 (0) | 0.1(0) | 1 (0) | -0.1(0) | 1.5 (0.7) | 0.3(0) | 1 (0) | 0.6(0) | | 1 (0) | 0.2(0) |
| ADAS Q4 Delayed word recall | | 8 (1.5) | 0.5(0) | 10 (0) | 0(0) | 9 (1.5) | 0.4(0) | 10 (0) | 0(0) | 9 (1.5) | 0.3(0) | | 8 (0) | 1.2(0) |
| ADAS Q5 Naming objects and fingers | | 0 (0) | 0.1(0) | 1 (0) | 0.4(0) | 0 (0) | 0.3(0) | 0.5 (0.7) | 1.2(0) | 0.5 (0.7) | 0.4(0) | | 0 (0) | 0(0) |
| ADAS Q6 Ideational praxis | | 0 (0) | 0.2(0) | 0 (0) | 0.6(0) | 0 (0) | 0.2(0) | 0 (0) | 0.7(0) | 0.5 (0.7) | 0.7(0) | | 0 (0) | 0.4(0) |
| ADAS Q7 Orientation | | 1 (1.5) | 0.4(0) | 2 (1.5) | 1.1(0) | 2 (1.5) | 1.2(0) | 3.5 (1.5) | 0.8(0.1) | 1 (0) | 2(0) | | 2 (1.5) | 1.1(0) |
| ADAS Q8 Word recognition | | 6 (3) | 0.6(0) | 7 (3) | 1.2(0) | 8 (3) | 0.4(0) | 11 (0.7) | -0.4(0.1) | 7.5 (4.4) | 0.9(0.1) | | 4 (1.5) | 2.7(0) |
| ADAS Q9 Remembering test instructions | | 0 (0) | 0.1(0) | 0 (0) | 0.3(0) | 0 (0) | 0.1(0) | 0 (0) | 0.7(0.1) | 0 (0) | 0.9(0) | | 0 (0) | -0.2(0) |
| ADAS Q10 Language | | 0 (0) | 0.1(0) | 0 (0) | 0.2(0) | 0 (0) | 0.2(0) | 0 (0) | 0.2(0) | 0 (0) | -0.1(0) | | 0 (0) | 0.3(0) |
| ADAS Q11 Word finding difficulty | | 0 (0) | 0.4(0) | 0 (0) | 0.5(0) | 0 (0) | 0.4(0) | 1.5 (0.7) | 0.8(0.1) | 1.5 (1.5) | 0.1(0.1) | | 0 (0) | 0.5(0) |
| ADAS Q12 Comprehension of spoken language | | 0 (0) 0.1(0) | 0.1(0) | 0 (0) | 0.1(0) | 0 (0) | 0.1(0) | 0 (0) | 1.3(0.1) | 0 (0) | -0.4(0) | | 0 (0) | 0.1(0) |

The data are presented as median (median absolute distance) unless otherwise stated. CSF: cerebrospinal fluid, MMSE: mini mental state examination, CDR: Clinical Dementia Rating, ADAS: Alzheimer’s disease assessment scale, m0 = first visit, m24 = visit after 24 months. Annual changes in the cognitive assessment scales were estimated with linear regression (follow up data as predictor, two parameters and variance estimation). Standard errors of the estimated parameters are included in brackets. No statistical tests between groups are performed due of small sample sizes in some of the groups. CSF values are in pg/ml. For the cluster diffuse 3 (Typical AD) we report data only for baseline and 12-month follow up since the subjects dropped out from the study due to disease severity.
