# Supplementary material for: Fully bayesian longitudinal unsupervised learning for the assessment and visualization of AD heterogeneity and progression
Source: Aging (Albany NY). 2020 Jul 9;12(13):12622–47. doi: 10.18632/aging.103623 (PMC7377879; doi:10.18632/aging.103623)
Supplement: Supplementary Table 2 [file aging-12-103623-s002..docx]

|  | Number of cluster | | | | | | | | | | | | | | | | | | | | | |
| --- | --- | --- | --- | --- | --- | --- | --- | --- | --- | --- | --- | --- | --- | --- | --- | --- | --- | --- | --- | --- | --- | --- |
|  | 2 | | | 3 | | | 4 | | | 5 | | | 6 | | | 7 | | | | 8 | | |
| Simulation initial values | Criterion 1 | Criterion 2 | Criterion 3 | Criterion 1 | Criterion 2 | Criterion 3 | Criterion 1 | Criterion 2 | Criterion 3 | Criterion 1 | Criterion 2 | Criterion 3 | Criterion 1 | Criterion 2 | Criterion 3 | Criterion 1 | Criterion 2 | Criterion 3 | Criterion 1 | | Criterion 2 | Criterion 3 |
| Previous study | 26116,0 | 1 | 0,18 | 25338,6 | 1 | 0,12 | 25630,6 | 1 | 0,12 | 41803,2 | 0 | 0,03 | 25009,6 | 2 | 0,08 | 24763,4 | 6 | 0,08 | 24639,1 | | 5 | 0,08 |
| K-means | 25632,7 | 0 | 0,17 | 25585,4 | 0 | 0,14 | 25313,2 | 3 | 0,13 | 24878,3 | 2 | 0,13 | 24189,7 | 3 | 0,11 | 24269,7 | 6 | 0,09 | 24184,6 | | 4 | 0,09 |
| Hierarchical clustering | 25710,5 | 4 | 0,20 | 25692,4 | 3 | 0,17 | 25101,9 | 5 | 0,13 | 24833,1 | 1 | 0,11 | 24758,9 | 6 | 0,10 | 24406,0 | 3 | 0,09 | - | | - | - |
| Previous study (with noise) | 25984,2 | 0 | 0,18 | 25311,2 | 1 | 0,12 | 25340,3 | 5 | 0,13 | 25790,2 | 5 | 0,09 | 24983,0 | 7 | 0,08 | 24839,6 | 8 | 0,08 | 24634,5 | | 3 | 0,06 |
| K-means (with noise) | 25592,7 | 0 | 0,19 | 25577,4 | 0 | 0,17 | 25316,9 | 0 | 0,11 | 24888,2 | 0 | 0,13 | 24255,2 | 0 | 0,11 | 24312,1 | 0 | 0,09 | 24318,7 | | 0 | 0,08 |
| Hierarchical clustering (with noise) | 26288,8 | 0 | 0,22 | 25443,0 | 0 | 0,18 | 25246,4 | 0 | 0,11 | 24571,9 | 0 | 0,10 | 24473,5 | 0 | 0,10 | 24161,7 | 0 | 0,09 | 24408,9 | | 0 | 0,07 |
| Package default values | 25605,0 | 0 | 0,19 | 26352,7 | 0 | 0,08 | 47471,9 | 0 | 0,07 | 46386,7 | 0 | 0,04 | 42471,4 | 0 | 0,03 | 46843,7 | 0 | 0,15 | 46440,2 | | 0 | 0,06 |

Supplementary Table 2. Model assessment.

Criterion1: Deviance, criterion 2: Observations with low certainty, criterion 3: Percentage of chains with high autocorrelation. Seven different strategies were used to initialize the model and a set of three criteria were calculated from each simulation to assess the resulting model quality: 1) Deviance (the model deviance), 2) observations with low certainty (After the calculation of the highest posterior density (HPD) intervals of the individual component probabilities of a subject to belong to any cluster, those subjects for whom the lowest HPD probability was lower than 0.5 were summed. The concept behind this quality assessment method, is that if the components (clusters) estimated from the optimization process cannot express sufficiently most of the AD subjects then they are not representative of the AD subsamples and therefore they do not represent AD pathophysiology adequately), 3) Percentage of chains with high autocorrelation (the MCMC chains that were auto-correlated were summed to provide a measure of general convergence).
