# Supplementary material for: Fully bayesian longitudinal unsupervised learning for the assessment and visualization of AD heterogeneity and progression
Source: Aging (Albany NY). 2020 Jul 9;12(13):12622–47. doi: 10.18632/aging.103623 (PMC7377879; doi:10.18632/aging.103623)
Supplement: Supplementary Tables 3 and 4 [file aging-12-103623-s003..pdf]

SUPPLEMENTARY TABLES

Supplementary Table 3. List of cortical and subcortical ROIs that were included in the analysis.

Cortical regions (thickness)	Subcortical regions (volume)
Banks superior temporal sulcus	Thalamus-Proper
Caudal anterior-cingulate cortex	Caudate
Caudal middle frontal gyrus	Putamen
Cuneus cortex	Pallidum
Entorhinal cortex	Hippocampus
Fusiform gyrus	Amygdala
Inferior parietal cortex	Accumbens-area
Inferior temporal gyrus	
Isthmus–cingulate cortex	
Lateral occipital cortex	
Lateral orbital frontal cortex	
Lingual gyrus	
Medial orbital frontal cortex	
Middle temporal gyrus	
Parahippocampal gyrus	
Paracentral lobule	
Pars opercularis	
Pars orbitalis	
Pars triangularis	
Pericalcarine cortex	
Postcentral gyrus	
Posterior-cingulate cortex	
Precentral gyrus	
Precuneus cortex	
Rostral anterior cingulate cortex	
Rostral middle frontal gyrus	
Superior frontal gyrus	
Superior parietal cortex	
Superior temporal gyrus	
Supramarginal gyrus	
Frontal pole	
Temporal pole	
Transverse temporal cortex	
Insula cortex	

Supplementary Table 4. Individual images of outlier clusters and HPD uncertain group.

RID	Group	Sex	Age	Age at the AD onset	Years of Education	APGEN 1	APGEN 2	CSF Abeta 42	CSF Ptau 181	CSF Alpha synuclein	MMSE score	MMSE Pentagon
404	7	F	88	84	14	3	3	234	32	1,3	20	2
404	7	F	89	84	14	3	3	234	32	1,3	22	1
404	7	F	90	84	14	3	3	234	32	1,3	20	1
1341	8	F	72	68	12	3	4	136	37	1,0	24	1
1341	8	F	73	68	12	3	4	136	37	1,0	22	1
1341	8	F	74	68	12	3	4	136	37	1,0	23	1
724	8	M	79	77	20	3	3	143	45	1,0	21	2
724	8	M	80	77	20	3	3	143	45	1,0	19	2
724	8	M	81	77	20	3	3	143	45	1,0	6	2
1281	HPD	F	78	68	16	4	4	94	41	0,7	25	2
1281	HPD	F	79	68	16	4	4	94	41	0,7	24	1
1281	HPD	F	80	68	16	4	4	94	41	0,7	21	1
753	HPD	M	65	63	16	4	4	129	62	4,2	24	1
753	HPD	M	66	63	16	4	4	129	62	4,2	20	1
753	HPD	M	67	63	16	4	4	129	62	4,2	17	1
852	HPD	F	84	83	18	3	4	131	32	NA	24	1
852	HPD	F	85	83	18	3	4	131	32	NA	22	1

The variable group here has three different values: 1 = Cluster 1, 7= cluster 7, HPD = High posterior density interval uncertain classification. APGEN 1 and 2 refer to the Apoe E4 alleles (3 is an Apoe E3 carrier and 4 is and Apoe E4 carrier). CSF values are in pg/ml.
